# The validity of socioeconomic status measures among adolescents based on self-reported information about parents occupations, FAS and perceived SES; implication for health related quality of life studies

**DOI:** 10.1186/s12874-016-0148-9

**Published:** 2016-04-29

**Authors:** P. Svedberg, J. M. Nygren, C. Staland-Nyman, M. Nyholm

**Affiliations:** School of Health and Welfare, Hamstad University, SE-301 18 Halmstad, Sweden

**Keywords:** Adolescents, Completion rate, Concurrent validity, Convergent validity health related Quality of Life (HRQOL), Measurement, Socioeconomic status

## Abstract

**Background:**

Research has shown inconsistencies in results and difficulties in conceptualization of assessment of socioeconomic status (SES) among adolescents. The aim of this study was thus to test the validity of self-reported information on SES in two age-groups (11–13 and 14–16 years old) in an adolescent population and to evaluate its relationship to self-reported health related quality of life (HRQOL). Different measures of SES commonly used in research in relation to HRQOL were tested in this study; parent’s occupations status, family material affluence status (FAS) and perceived SES.

**Method:**

A cross-sectional study, with a sample of 948 respondents (*n* = 467, 11–13 years old and *n* = 481, 14–16 years old) completed questionnaires about SES and HRQOL. The adolescents’ completion rates were used, with chi2-test, to investigate differences between gender and age-group. Correlation was used for *convergent validity* and ANOVA for *concurrent validity*.

**Results:**

We found a low completion rate for both fathers’ (41.7 %) and mothers' (37.5 %) occupation status, and a difference in completion rate between gender and age-groups. FAS had the highest completion rate (100 %) compared to parent's occupations status and perceived SES. The convergent validity between the SES-indicators was weak (Spearman correlation coefficient below 0.3), suggesting that the indicators measured different dimensions of SES. Both FAS and perceived SES showed a gradient in mean HRQOL between low and high SES in relation to HRQOL, this was significant only for perceived SES (*p* < 0.01, both age-groups).

**Conclusion:**

This study indicates the need for considering different approaches to measures of SES among adolescences and when evaluating SES in relation to HRQOL. Further research is needed to investigate sustainable ways to measure SES, delineating the relevance of tangible measures of education, occupation and income in relation to the perceived socioeconomic status in comparison with others in immediate social networks and in society at large.

## Background

Socioeconomic status (SES) is an important determinant of physical, psychological and social development and of inequalities in health related quality of life (HRQOL) among adolescents [1–6]. Adolescents’ with low socioeconomic status are more likely to have poorer health outcomes compared to adolescents from groups with higher socioeconomic status [7–10]. Research has shown that SES among adolescent differ between age and gender [11, 12] but the SES gradient in health outcomes among adolescents’ is inconsistent [13]. Among adults, the socioeconomic-gradient in HRQOL is well established, despite using different measures of SES ([13–15], Goodman et al. 2001). This inconsistency in SES gradient in health outcomes among adolescents could be explained by methodological and measurement issues [10].

Self-reported SES among adolescents’ is not commonly included in health and medical research. Instead the relationship between SES and HRQOL is often derived from SES measures based on parents’ education and occupation [7, 16–18], with information collected by parents or adolescent themselves. However, parents’ education and parents’ occupation as measures of SES have been debated as not being suitable proxies for self-reported SES among adolescents [4, 15, 19, 20]. This is due to potential disagreement between adolescents and proxy-respondents’ report of education and occupation, high level of non-responders among adolescents’ and hence uncertainties about the validity and meaning of the measurements [4, 15, 19]. Another approach is to measure family wealth and material affluence as a marker for socioeconomic status [9, 21, 22]. The Family Affluence Scale (FAS) measures material affluence and contains quantitative questions about the ownership of computers and cars, family holidays and having a private room in their home, which is aggregated to attain a sum score. This FAS-score has been shown to be associated with the parental social class [22]. Another way to measure SES among adolescents is by using the subjective ratings of social status, i.e. their perceived position within the social economic hierarchy within the population or community. Subjective SES can be measured through questions about adolescents’ perception of their family’s socio-economical, financial or social status [10, 23]. The most common measurements are using a 10-point ladder (for example MacArthur Scale) or a Likert scale (for example HBSC) to investigate subjective SES [13, 24, 25]. Measuring the subjective SES of adolescents’ seems to be less dependent on the use of specific measurements or scales, instead similar results has been yielded in relation to health outcomes [10]. There is a need for more research regarding the correlation between different types of SES measurements in adolescents and the validity of each of these instruments in order to increase the understanding about the pathways between SES and HRQOL The aim of this study was to explore the validity of different types of measurements of self-reported information on SES in two age-groups (11–13 and 14–16 years old) in a Swedish adolescence population. Furthermore, we investigated how these SES measures related to levels of mean self-reported HRQOL.

## Methods

### Study population

This cross-sectional study is based on data from the Halmstad Youth Quality of Life cohort collected in 2011 in Sweden. The sampling frame included pupils from all municipal schools (*n* = 7) that were centrally located in the city of Halmstad and each having a total of more than 100 pupils. In total 50 classes were invited to participate in the study, resulting in a sample of 24 classes with pupils 11–13 years old (*n* = 536 pupils, younger age group) and 25 classes with pupils 14–16 years old (*n* = 576 pupils, older age group). One class (14–16 years) decided not to participate. A sample of 948 respondents (*n* = 467 11–13 years old and *n* = 481 14–16 years old) completed the questionnaires yielding a response was rate of 87 and 84 % respectively (non-respondents were almost exclusively due to absence from school during the data collection). Adolescents answered a self-report questionnaire consisting of the Minneapolis Manchester Quality of Life instrument, (MMQL) and questions about socioeconomic status.

The principal at each school approved participation in the study. Prior to the data collection, the schools distributed written information to children and their parents about the purpose of the research and that participation was voluntary and that if the children or the parents declined participation, they could decide to not fill in the questionnaire without having to explain any reasons why. Questionnaires were distributed to each class following a brief introduction by the researchers (PS; JN) and with a continued possibility to ask questions about the study or specific questions during completion of the questionnaire. Completed questionnaires were returned by each respondent and collected by the researchers (PS; JN), except for two schools where teachers distributed and collected the questionnaires in return envelopes from each class. A total of 210 adolescents were excluded due to missing data and 741 adolescents participated in this study.

### Measurements

#### Socioeconomic status (SES)

##### Parent’s occupation status

Parents occupation was based on the occupational status of the mother and father reported by the adolescents. The questions were; “Do you know what your father works with?” and “Do you know what your mother works with?” Adolescents whose answer to the question, were either not interpretable such as “at a company, a name of a shop or just at the Hospital” or blank, were analyzed together as missing data. Answers such as; “nurse at the hospital” or “police”, were categorised according to the Swedish socioeconomic classification index (SEI) – standardized by Statistics Sweden [26]. The SEI-groups were combined into five categories; high-level non-manual employees, medium level non-manual employees, low-level non-manual employees, skilled manual workers, unskilled manual workers and unemployed [26, 27]. The five categories were in this study further categorised into manual (white collar), non-manual (blue collar) and unemployed [28].

##### *FAS-* score

The Family Affluence Scale (FAS), used in this study, is one of the most common tools or standardized measurements to measure the objective SES of adolescents [22]. It was developed by the World Health Organization (WHO) as a measure of family wealth and comprises four items including; parental car ownership (“Does your family own a car, van, or a truck?” (0, 1, 2)), sharing or not sharing bedroom (“Do you have your own room?” (1, 0)), number of holidays per year (“During the past 12 months, how many times did you travel away on holiday with your family?” (0, 1, 2, 3)), having computers at home (“How many computers does your family own?” (0, 1, 2, 3)) [22]. The composite FAS score was calculated for each adolescent by adding of the four items (ranging from 0–9) and further categorised into low (0–5), medium (6–7) and high (8–9) [29].

##### Perceived SES

The perceived SES is a measurement that captures adolescents perception of their family’s socioeconomical, financial or social status [10, 23]. Perceived SES was measured with a question from the HBSC survey [30]; “How well off do you think your family is?” with five response alternatives; “not at all”, “not particularly”, “fairly”, “rather” and “very”. The five response alternatives were clustered into three categories; low (“not at all” and “not particularly”), middle (“fairly”) and high (“rather” and “very”).

#### Quality of life

##### Minneapolis Manchester Quality of Life instrument, (MMQL)

MMQL is a self-assessment instrument available in two age-appropriate versions, the MMQL-Youth form for children aged 8–12 years old and the MMQL-Adolescent form for children aged 13–20 years old [31, 32]. Both instruments have good psychometric properties and are available in versions translated and validated in a Swedish context [23, 24]. The MMQL-Youth form consists of four quality of life subscales; physical symptoms, physical functioning, psychological functioning and outlook on life/family dynamics divided into 32 items. The MMQL-Adolescent form consists of seven quality of life subscales; physical functioning, cognitive functioning, psychological functioning, body image, social functioning, intimate relations and outlook on life divided into 45 items. The responses to each item are given with a Likert-type scale from never to always: “Never” = 5; “Seldom” = 4, “Sometimes” = 3, “Mostly” = 2 and “Always” = 1. All item scores were aggregated and then divided with the number of item derive a total score of health related quality of life.

### Statistical analysis

All analyses were performed using the whole sample and some analyses were repeated after stratifying for age group (11–13 years and 14–16 years) and gender. Statistical analyses were conducted using SPSS PC version 14.0. Statistical significance in all analyses was determined at *P* < 0.05. The general characteristics of the sample was described using mean and standard deviation (SD), numbers (n) and proportions (%). The response rates and rates of missing items for parent’s occupation status, FAS and perceived SES were described using numbers (n) and proportions (%). Differences in the rates of missing items between gender and age-groups were analysed using the chi^2^- test. Validity was tested with the following criteria [33, 34]: for *convergent validity* the computed Spearman correlation coefficient was applied between levels (low, medium and high) of parent’s occupations, FAS and perceived SES. Spearman correlation coefficients below 0.30 were considered weak, 0.30 to 0.59 moderate and ≥ 0.60 high [25]. *Convergent validity* was considered to be achieved when correlations were moderate or high [25]. For *concurrent validity* ANOVA was used to test the ability of the SES measures to yield parents occupations status (manual, non-manual and un-employed) and levels low, mediate and high of FAS and perceived SES in relation to the mean of the total-MMQL score [26].

## Results

Of the 741 study participants, 50.1 % were girls in the younger age group and 47.8 % in the older age group. The mean (standard deviation) MMQL-score were 4.34 (0.37) in the younger age-group and 4.01 (0.42) in the older age group (Table 1).

**Table 1:** General characteristics in 11–13 years old and 14–16 years old pupils participating in the Halmstad Youth Cohort (*n* = 741)

	Age-group			
	11–13 years old		14–16 years old	
	(*n* = 377)		(*n* = 364)	
	mean	(SD)	mean	(SD)
*HRQoL (total)*	4.34	(0.37)	4.01	(0.42)
	*n*	(%)	*n*	(%)
*Gender*				
Boys	188	(49.9)	190	(52.8)
Girls	189	(50.1)	174	(47.8)
*Parents occupation status*				
*Fathers occupation status*				
Non-manual workers (white collar)	101	(50.2)	117	(51.3)
Manual workers (blue collar)	91	(45.3)	103	(45.2)
Unemployed	9	(4.5)	8	(3.5)
*Mothers occupation status*				
Non-manual workers (white collar)	140	(65.7)	159	(63.6)
Manual workers (blue collar)	58	(27.2)	75	(30.0)
Unemployed	15	(7.0)	16	(6.4)
*Fas-score*				
High (8–9 items)	111	(29.2)	95	(26.1)
Medium (6–7 items)	160	(42.4)	191	(52.5)
Low (0–5 items)	106	(28.1)	78	(21.4)
*Percieved SES*				
High	134	(36.7)	85	(24.5)
Medium	187	(51.2)	209	(60.1)
Low	44	(12.1)	54	(15.5)

Completion rates were lowest for parent occupations status, 41.7 % for fathers’ occupation status and 37.5 % for mothers’ occupations status (Table 2). There was a significant difference between the age groups, where the youngest age group had the highest proportion of non-responders compared to the older age group, mothers’ occupation status (*p* = 0.001) and fathers’ occupation status (*p* = 0.012). Girls had a higher completion rate than boys for the question on mothers’ occupation status (*p* = 0.004) and on fathers’ occupation status (*p* = 0.015). No such difference was seen for the perceived SES (Table 2).

**Table 2:** Completion rate in parents occupation status, FAS and percieved SES

	Fathers occupation status			Mothers occupation status				FAS-score		Percieved SES		
	Respondent	Non-respondent	p*	Respondent	Non-respondent	p*	Respondent	Non-respondent	p*	Respondent	Non-respondent	p*
	*n* (%)	*n* (%)		*n* (%)	*n *(%)		*n* (%)	*n* (%)		*n* (%)	*n* (%)	
*Total*	432 (58.3)	309 (41.7)		463 (62.5)	278 (37.5)		741 (100)			713 (96.2)	28 (3.8)	
*Age-group*												
11–13 years	201 (53.3)	176 (46.7)	0.012	210 (55.7)	167 (44.3)	0.001	377 (100)	-	-	365 (96.8)	12 (3.2)	0.387
14–16 years	224 (61.5)	140 (38.5)		252 (69.2)	112 (30.8)		364 (100)	-	-	348 (95.6)	16 (4.4)	
*Gender*												
Boys	204 (53.7)	174 (46.3)	0.015	217 (57.4)	161 (42.6)	0.004	378 (100)	-	-	363 (96.0)	15 (4.0)	0.782
Girls	228 (62.8)	135 (37.2)		246 (67.8)	117 (32.2)		363 (100)	-	-	350 (96.4)	13 (3.6)	

*P-value differenes in completion rate were analysed with CHI2 -test

Convergent validity was evaluated by Spearman’s correlation coefficient between the categorized levels of parents’ occupational status, FAS and perceived SES (Table 3). The highest significant correlation coefficient in the younger age group was seen between mothers’ and fathers’ occupational status *r* = 0.303 (*p* < 0.001) suggesting that these measures are measuring the same construct in this age group. This was not seen in the older age group, where the highest correlation coefficient was seen between FAS and perceived SES, the correlation was, however, weak (*r* = 0.292, *p* < 0.001) (Table 3). All other correlations in both age groups were considered weak (*r* below 0.300), suggesting that these indicators measure different dimensions of self-rated SES.

**Table 3:** Convergent validity - Spearman correlations coefficient between parents occupation status, FAS and percieved SES

Measure	Fathers occupation status	Mothers occupation status	FAS-score	Percieved SES
			14–16 years old	
Fathers occupation status	**1**	**0.225**	**0.197**	0.113
Mothers occupation status	**0.303**	**1**	**0.199**	0.083
FAS-score	**0.184**	**0.246**	**1**	**0.292**
Percieved SES	**0.143**	0.063	**0.262**	**1**
		11–13 years old		

*Boldface indicates P<0.05*

For concurrent validity, each SES measure was used to differentiate between mean self-reported HRQOL of those having parents with manual, non-manual and unemployed occupational status and having high, medium or low levels of SES in FAS and perceived SES (Table 4). The analysis of validity showed no gradient of HRQOL when using fathers’ and mothers’ occupational status whereas when using FAS to grade the mean HRQOL the lowest level of mean HRQOL was found for those with lower SES. This analysis was neither significant in the younger age group (*p* = 0.052) nor in the older age group (*p* = 0.065), however, the perceived SES showed a significant gradient of mean HRQOL in the different levels of SES in both age-groups *p* < 0.001 (Table 4).

**Table 4:** Concurrent validity - comparison of HRQoL total score in parents occupation status, FAS and percieved SES

	Age-group			
	11–13 years old		14–16 years old	
	HRQol total score		HRQol total score	
	mean	(SD)	mean	(SD)
Fathers occupation status	(*n* = 192)		(*n* = 219)	
Non-manual workers (white collar)	4.33	(0.35)	4.05	(0.38)
Manual workers (blue collar)	4.29	(0.41)	3.99	(0.46)
Unemployed	4.47	(0.47)	3.90	(0.39)
*p-value*	*0.996*		*0.171*	
Mothers occupation status	(*n* = 198)		(*n* = 233)	
Non-manual workers (white collar)	4.33	(0.39)	4.03	(0.40)
Manual workers (blue collar)	4.34	(0.39)	4.00	(0.43)
Unemployed	4.24	(0.37)	3.91	(0.38)
*p-value*	*0.517*		*0.246*	
FAS-score	(*n* = 377)		(*n* = 365)	
High	4.41	(0.34)	4.06	(0.39)
Medium	4.31	(0.38)	4.03	(0.41)
Low	4.30	(0.39)	3.92	(0.47)
*p-value*	*0.052*		*0.065*	
Percieved SES	(*n* = 365)		(*n* = 347)	
High	4.41	(0.36)	4.10	(0.42)
Medium	4.35	(0.35)	4.04	(0.36)
Low	4.07	(0.45)	3.77	(0.56)
*p-value*	*<0.001*		*<0.001*	

## Discussion

### Principal findings

We found a low convergent validity between the SES indicators, where weak correlations were found between parents’ occupational status, FAS and the perceived SES, suggesting that these indicators measure different reflections of SES among adolescents. In terms of concurrent validity we found that HRQOL, in relation to both FAS and perceived SES, showed a gradient between mean HRQOL, however this was significant only for perceived SES. Now such relation was seen when using measures of parents’ occupational status.

Results from this study showed low convergent validity between the three different SES indicators, which confirms that there are difficulties in relying on only one SES indicator when measuring adolescents’ SES. This is especially true when parents’ occupational status is used as a proxy for self-reported SES among adolescents’ [20, 34]. In line with earlier studies, difficulties in the assessment of parents’ occupation (i.e. parents’ socio-economic status) were found. There were respondents, in both age groups, who had difficulties in specifying parents’ occupation and were unable to answer this question which raise questions in terms of validity [35]. The proportion of non-responses was higher in the younger age group and boys were more likely than girls to be non-responders. Difficulties for young people in assessing parents’ occupation status may be due to re-call bias but probably also to the fact that contemporary working life comprises a variety of different types of work tasks and work places that may be difficult for children of younger ages to comprehend. For a smaller group of children and adolescents, non-responses may also reflect feelings of shame due to parents being unemployed or long-term sick which respondents may not consider to be socially acceptable [4].

FAS, on the other hand, reflect tangible material assets on a daily basis which is likely to explain the high completion rate. Despite the high completion rate and the widespread use of the FAS scale as an indicator of family SES in surveys with high face validity, there are limitations that need to be considered. In this study the FAS scale did not show any significant association with HRQOL. Since the development of FAS the average material standard in society has increased. It might be that material standard measured in terms of numbers of computers, bedrooms, cars and holiday trips does not sufficiently reflect socio-economic inequalities and thus can not adequately differentiate between degrees of material affluence in different groups of adolescents [22]. This could in particular be the case in a country that, in an international comparison, is characterized by a high economic and technological standard and a compressed income structure, particularly if there is a developed welfare system with general support systems aiming to reduce economic disparities in the society entailing less material difference between those well off and those less well off. It is likely that FAS can reveal differences in material standard between high socio-economic status groups versus economically deprived groups but less suitable for detecting differences in midrange groups. Since Sweden, in international surveys, stands out as a country were severe material deprivation is rare the inability of FAS to distinguish between socioeconomic groups could be somewhat questioned and our results therefore needs to be interpreted carefully [36]. It is also important to notice that the assessment of numbers of computers in a home may not always mirror an explicit affluence standard since certain companies or employers provide their staff with possibility to use office computers at home or provide home computers with reduced costs. Moreover, it is possible that a comprehensive introduction of various mobile devices in many respects replace the need for home computers. Another reason for the non-significant association to HRQOL may be found in the construction of the scale where FAS does not explicitly meet the terms for SES (education, occupation, income). Respondents can achieve their score on FAS in different ways not always closely connected to their parents’ absolute SES or affluence standard. High scores on FAS can be reported by young people in high-income families who can afford several cars, computers and holiday trips but the same FAS score may also be reported by an adolescent in a low-income family where material possessions are bought with for example loans. On the other hand, adolescents scoring *not having a car* may live in a well-off family with a centrally located apartment in a city with good public transportation. Even though this will generate a lower FAS score it does not reflect a lower material standard and in this case the validity of FAS will be adverse.

The perceived SES was the only significant indicator that showed a significant gradient of mean HRQOL in the different levels of SES in both age groups. Earlier studies have discussed that SES is a multidimensional construct and it is important to understand how the construct of subjective SES is conceptualized and understood among adolescents. In contrast to the affluence scale, perceived SES measures adolescents’ perception of their everyday life in terms of economic situation, and thus a wider concept of perceived social status. It has earlier been discussed that perceived social status not only assesses the situation at a certain time point but also captures a cumulative influence of different experiences throughout the life span adding to a person’s perceived relative position in the social hierarchy [37, 38]. Perceived SES is understood to assess more salient dimensions of adolescents’ perceptions of their social status than more objective measures (i.e. parents’ occupation status), such as their immediate social interactions with their closest and most important environment [39]. Earlier studies have also discussed that perceived SES includes a psychological dimension, not only objective resources, closely related to perceived social status. When assessing wealth in one’s family (i.e. social status) in comparison with others’ it is likely that adolescents refer to both proximal referents e.g. perceived richness in friends and class-mates and distal referent social groups e.g. comparisons with young people or other families in the society [40]. Due to the immense development of boundless Internet based social communication it is likely that there is an increased impact of referent groups on adolescents’ views about richness which influence perceptions of family wealth and position in the social hierarchy. In a review, perceived SES has been shown to predict health outcomes better than traditional measurements [41]. The perception of relative social position has also been suggested to mediate the association between economic inequality and material standard and population health [42]. With this understanding, perceived SES may be a more sensitive socio-economic measure than objective SES indicators (i.e. parents’ occupational status) in adolescents, which could be part of the explanation for the significant results on HRQOL in this study.

### Strengths and limitation

A strength of this study was that the data was based on a sample of young people within their period of adolescenthood. To be able to explore possible associations between SES and HRQOL and to generate further age-related hypotheses, two age groups (11–13 and 14–16 years of age) were chosen. However, the data was a non-probability sampling, with an assumption of an even distribution of the characteristics within the population. A limitation of this might be the difficulties to identify all possible biases. The instruments used in this study are well established, internationally accepted and commonly used measures of SES and HRQOL. The key limitations of this study were the cross-sectional design and that the analyses were based only on self-rated data of adolescents. In this study, parents’ occupation status was classified into three categories in the analyses, according to the Swedish socioeconomic classification index [26]. This categorization could be a limitation as it might be too rough and as it does not capture all levels of the parents’ occupational status in the area such as in distinction between high, medium, and low non-manual employees and between skilled and unskilled manual workers. All component items in the FAS scale were given an equal weight in this study. It is possible that this way of handling the analysis reduced the scale’s ability to discriminate between different components. It has been discussed in work of Batista-Fouget and colleagues (2004) that different weights should be applied to the different items depending on their expected importance in different countries [31]. However, due to the considerations concerning the FAS scale highlighted above we do not believe that this was the decisive reason behind the results on the FAS scale in this study. Perceived SES in previous studies have been measured in different ways [10, 13, 23–25], however this concept need to be more discussed and further investigated in face and content validity among adolescents between 11–16 years.

## Conclusion

In conclusion, there was a low concurrent validity between the traditional indicators of SES, FAS and the subjective SES in this adolescent population. This might indicate that self-rated information on SES measures different dimensions of SES among adolescents. However, in relation to HRQOL, only subjective SES showed a significant gradient between the mean HRQOL of low and high SES. It can be suggested that subjective SES might measure a wider construct of perceived social status, where social networks and popularity have a stronger impact on adolescence HRQOL. It can also be implied from our findings that different approaches to measures of SES among adolescents should be considered when evaluating SES, at individual and family level as well as at area levels, in relation to HRQOL. Further research is needed to investigate sustainable ways to measure SES, delineating the relevance of tangible measures of education, occupation and income in relation to the perceived socioeconomic status in comparison with others in immediate social networks and in society at large.

### Ethics and consent statement

The Regional Ethical Review Board in Lund, Sweden (Dnr 2013/55) formally approved the study. The participants and their guardians were given written and oral information about the aim of the study, what participation entailed, the confidentiality of the data and that they had right to withdraw from the study at any time. Consent was obtained from all participants and their guardians.

### Availability of data and materials

The dataset supporting the conclusions of this article are available from the corresponding author on request.

## Abbreviations

FAS: family material affluence status
FOB 85: OCCUPATIONS in population and housing census 1985
HBSC: health behaviour in school-aged children
HRQOL: health related quality of life
MMQL: Minneapolis Manchester quality of life instrument
NYK: Nordicordic standard occupational classification
SCB: Stockholm Statistiska Centralbyrå Statistics-Sweden
SEI: the Swedish socioeconomic classification index
SES: socioeconomic status
SPSS: Statistical Package for Social Sciences
